# Dynamic Rearrangement of F-Actin Is Required to Maintain the Antitumor Effect of Trichostatin A

**DOI:** 10.1371/journal.pone.0097352

**Published:** 2014-05-20

**Authors:** Dong-Hee Yang, Jae-Wook Lee, Jiyoung Lee, Eun-Yi Moon

**Affiliations:** Department of Bioscience and Biotechnology, Sejong University, Seoul, Republic of Korea; Institute of Pathology, Germany

## Abstract

Actin plays a role in various processes in eukaryotic cells, including cell growth and death. We investigated whether the antitumor effect of trichostatin A (TSA) is associated with the dynamic rearrangement of F-actin. TSA is an antitumor drug that induces hyper-acetylation of histones by inhibiting histone deacetylase. HeLa human cervical cancer cells were used to measure the antitumor effect of TSA. The percent cell survival was determined by an MTT assay. Hypodiploid cell formation was assessed by flow cytometry. Collapse of the mitochondrial membrane potential (MMP) was identified by a decrease in the percentage of cells with red MitoProbe J-aggregate (JC-1) fluorescence. Cell survival was reduced by treatment with TSA, as judged by an MTT assay and staining with propidium iodide, FITC-labeled annexin V, or 4′,6-diamidino-2-phenylindole (DAPI). TSA also induced an MMP collapse, as judged by the measurement of intracellular red JC-1 fluorescence. In addition, the F-actin depolymerizers cytochalasin D (CytoD) and latrunculin B (LatB) induced an MMP collapse and increased apoptotic cell death in HeLa cells. However, our data show that apoptotic cell death and the MMP collapse induced by TSA were decreased by the co-treatment of cells with CytoD and LatB. These findings demonstrate that the dynamic rearrangement of F-actin might be necessary for TSA-induced HeLa cell apoptosis involving a TSA-induced MMP collapse. They also suggest that actin cytoskeleton dynamics play an important role in maintaining the therapeutic effects of antitumor agents in tumor cells. They further suggest that maintaining the MMP could be a novel strategy for increasing drug sensitivity in TSA-treated tumors.

## Introduction

Actin microfilaments are cytoskeletal protein polymers that are critical for cellular processes such as growth, motility, division, and apoptosis [1], [2]. Changes in the dynamics of the actin cytoskeleton may lead to cell death [3]. The drugs jasplakinolide (JasPK), cytochalasin D (CytoD), and latrunculin B (LatB) induce major changes in microfilament dynamics [4], [5]. JasPK stabilizes the actin cytoskeleton and induces the accumulation of large filamentous (F)-actin aggregates [6], [7]. CytoD promotes nucleation and causes the complete collapse of stress fibers [4], [5]. LatB causes shortening and thickening of stress fibers by forming a nonpolymerizable complex [4], [5]. Actin or actin-binding proteins can influence mitochondrial pathways [8]. Increased amounts of gelsolin were found to be co-localized with actin stress fibers and distributed in the nucleus and mitochondria in senescent human diploid fibroblasts [9]. ADF/cofilin family proteins are also essential regulators of actin cytoskeletal dynamics and regulate both mitochondrial function and stress responses in the budding yeast *Saccharomyces cerevisiae* [10]. Mitochondrial dysfunction causes alterations in cellular morphology and adhesion [11], and mitochondria-actin interactions contribute to cell death [8].

Changes in the turnover of F-actin seem to trigger cell death through an apoptosis-like pathway [3], [12]–[14]. CytoD increases apoptosis in human CMK-7 cells [15], while JasPK increases apoptosis in mammalian cells [6], [7]. In addition, down-regulation of the actin-severing protein gelsolin stabilizes the actin cytoskeleton and increases apoptosis [16]. By contrast, CytoD protects gelsolin-deficient cells from apoptosis [16]. While CytoD was shown to have no effect on basal apoptosis, it attenuated apoptosis during ischemia-reperfusion in human umbilical vein endothelial cells [17]. This suggests that cytoskeletal dynamics are involved not only in tumor cell death but also in drug resistance induction. However, little is known about whether drug resistance can be induced by treatment with antitumor therapeutics such as the histone deacetylase (HDAC) inhibitor trichostatin A (TSA) through changes in cytoskeletal dynamics.

Anticancer treatment is hampered by the resistance of tumor cells to chemotherapy, which leads to decreases in tumor patient survival rates [18]. A tumor’s surrounding tissues and environment can be acutely altered by treatment with chemotherapeutics, and this can contribute to drug resistance in tumor cells. In addition, changes in intracellular molecules play a role in drug resistance. For example, the overexpression of glucose-regulated protein 78 kDa (GRP78/BiP) induces resistance to HDAC inhibitor-induced apoptosis in cancer cells. Conversely, the suppression of GRP78 sensitizes cells to HDAC inhibitors [19]. GRP78/BiP also preserves the mitochondrial membrane potential (MMP) after stress [20]. However, little is known about the role of changes in the MMP in TSA resistance.

Here, we investigated whether resistance to the HDAC inhibitor TSA could result from changes in cytoskeletal dynamics through a collapse of the MMP. Our data show that TSA and F-actin depolymerizers induced an MMP collapse and increased apoptotic cell death in HeLa cells. Our data also show that the co-treatment of HeLa cells with CytoD and LatB reduced apoptotic cell death and the MMP collapse induced by TSA. These data suggest that the prevention of F-actin rearrangements by CytoD and LatB treatment can decrease the TSA sensitivity of tumor cells through the inhibition of a further MMP collapse.

## Materials and Methods

### Reagents

3(4,5-Dimethyl-thiazol-2-yl)-2,5-diphenyl tetrazolium bromide (MTT) and propidium iodide (PI) were purchased from Sigma Chemical Co. (St. Louis, MO). Phalloidin-tetramethyl-rhodamine B isothiocyanate (TRITC) was obtained from Molecular Probes (Eugene, OR). MitoProbe J-aggregate (JC-1; 5,5′,6,6′-tetrachloro-1,1′,3,3′-tetraethyl-benzimidazolyl-carbocyanine iodide) and 4′,6-diamidino-2-phenylindole (DAPI) were purchased from Life Technologies (Grand Island, NY). An annexin V apoptotic cell detection kit was purchased from eBioscience Inc. (San Diego, CA). Except where indicated, all other materials were obtained from Sigma Chemical Co.

### Cell Culture

HeLa cells were obtained from the Korea Research Institute of Bioscience and Biotechnology cell bank (Daejeon, Korea). Cells were maintained and cultured in Dulbecco’s modified Eagle’s medium supplemented with 10% fetal bovine serum (Hyclone, Kansas City, MO), 2 mM L-glutamine, 100 U/ml penicillin, and 100 U/ml streptomycin.

### MTT Assay

We quantified cell survival using a colorimetric assay that measured the intracellular succinate dehydrogenase content using MTT [21]. Confluent cells were cultured with various concentrations of each reagent for 48 h. The cells were then incubated with 50 µg/ml MTT at 37°C for 2 h. The formazan product was dissolved in dimethyl sulfoxide. The optical density at 595 nm was then measured.

### Flow Cytometric Analyses

For the determination of hypodiploid cell formation, cells were fixed in 40% ethanol on ice for 30 min and then incubated with PI (50 µg/ml) and RNase (25 µg/ml) at 37°C for 30 min. In addition, cells were stained with annexin V-FITC and PI to analyze early and late apoptotic cells. The stained cells were analyzed using CELLQuest software and a FACSCalibur flow cytometer (Becton Dickinson, San Jose, CA).

### Measurement of the MMP

For each sample, cells were suspended in 1 ml of PBS buffer at ∼1×10^6^ cells/ml. JC-1 (final concentration, 2.5 µg/ml) was added to the sample, which was incubated for 10 min at 37°C. The stained cells were then centrifuged at 400×*g* for 5 min at room temperature and the supernatant was removed completely without disturbing the cell pellet. The pellet was then washed with 1–2 ml of PBS. The cells were analyzed immediately with a NucleoCounter NC-3000 cytometer (ChemoMetec, Alleroed, Denmark). Cellular green and red fluorescence was quantified and cells with collapsed MMPs exhibited a decrease in the red/green fluorescence intensity ratio.

### Immunostaining

F-actin was detected by immunostaining. HeLa cells were plated on cover glasses then incubated with each reagent for the appropriate amount of time. They were then fixed in 3% paraformaldehyde before being stained with DAPI and/or phalloidin-TRITC diluted 1∶1,000 in sucrose buffer (10 mM HEPES, 3 mM MgCl_2_, 50 mM NaCl, 300 mM sucrose, and 0.5% Triton X-100). The cells were observed at 400× or 1,000× magnification under a fluorescence microscope (Nikon, Tokyo, Japan).

### Statistical Analyses

Experimental differences were tested for statistical significance using an ANOVA and Student’s *t*-test. A *p*-value of <0.05 was considered to be significant.

## Results

### TSA-induced Cell Death was Inhibited by Treatment with CytoD or LatB

**Figure 1:**
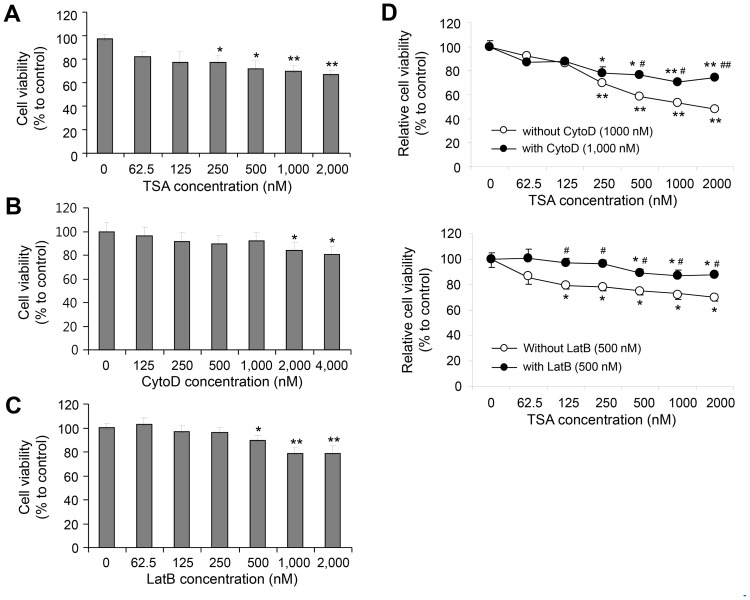
TSA-mediated cell death in HeLa cells was inhibited by co-treatment with CytoD or LatB. Hypodiploid cell formation was increased by incubation with TSA, CytoD, or LatB. **A–C:** HeLa cells were treated with various concentrations of TSA (A), CytoD (B), or LatB (C) for 48 h. **D:** HeLa cells were treated for 36 h with various doses of TSA (•) in the presence (○) or absence of CytoD (upper) or LatB (lower). Cell density was measured by an MTT assay, as described in the Materials and Methods. The data in the line graph represent the mean ± SEM. *p<0.05, **p<0.01 vs. non-TSA-treated and non-CytoD- or non-LatB-treated control cells (A**–**E). ^#^ *p*<0.05, ^##^ *p*<0.01 vs. TSA-treated and non-CytoD-treated (D) or non-LatB-treated (E) control cells.

To test the antitumor effect of TSA, we measured cell viability by an MTT assay. When cells were treated with 2 µM TSA for 48 h, cell viability was decreased by about 25% (Figure 1A). To test whether the TSA-induced changes in cell viability involved cytoskeletal dynamics, cells were treated with the actin cytoskeleton depolymerizers CytoD and LatB. CytoD and LatB also reduced cell viability (Figure 1B and C): 4 µM CytoD and 2 µM LatB decreased cell survival by about 10 and 20%, respectively. Next, we examined whether treatment with CytoD or LatB would affect TSA-induced changes in cell viability. TSA-induced cell death was significantly inhibited by co-treatment with 1,000 nM CytoD (Figure 1D, upper) or 500 nM LatB (Figure 1D, lower) for 36 h. This suggests that cell viability was significantly increased by treatment with TSA in the presence of CytoD as compared to that in CytoD-treated and non-TSA-treated control cells (Figure 1D, upper). In addition, cell viability was significantly increased by treatment with TSA in the presence of LatB as compared to that in LatB-treated and non-TSA-treated control cells (Figure 1D, lower). This suggests that F-actin rearrangement is necessary for TSA-induced HeLa cell apoptosis.

**Figure 2:**
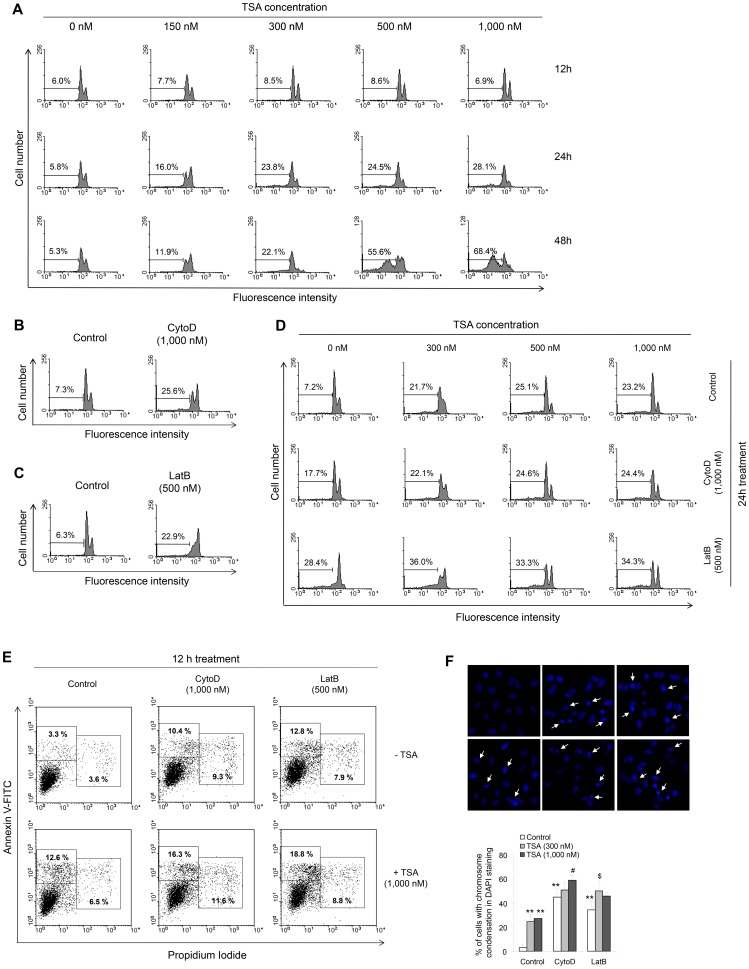
Hypodiploid cell formation in HeLa cells was inhibited by treatment with CytoD or LatB in the presence of TSA. **A:** HeLa cells were treated with various concentrations of TSA for 12, 24, or 48**B:** HeLa cells were treated with 1,000 nM CytoD for 6, 12, or 18 h. **C:** HeLa cells were treated with 500 nM LatB for 3, 6, or 12 h. **D:** HeLa cells were treated for 24 h with various concentrations of TSA in the presence or absence of 500 nM LatB or 1,000 nM CytoD. Cells were fixed with 40% ethanol and stained with PI, and then hypodiploid cells were analyzed by flow cytometry. **E–F:** HeLa cells were treated for 16 h with 1,000 nM TSA in the presence or absence of 500 nM LatB or 1,000 nM CytoD. Cells were stained with annexin V-FITC and PI, and then analyzed by flow cytometry (E). Cells were fixed with 3% paraformaldehyde and stained with DAPI, and then observed under a fluorescence microscope at 400× magnification. Arrows indicate representative cells with chromosome condensation in each group (F, left). The data in the bar graph represent the mean ± SEM. **p<0.01 vs. untreated control cells; ^#^ *p*<0.05 vs. TSA-treated and non-CytoD-treated control cells; ^$^ *p*<0.05 vs. TSA-treated and non-LatB-treated control cells (F, right).

Our findings were confirmed by an analysis of hypodiploid cell formation using TSA-treated cells. As shown in Figure 2A, the percentage of hypodiploid cells was time-dependently increased by treatment with various concentrations of TSA. The percentage of S-phase cells was decreased while the percentage of G2/M-phase cells was increased by incubation with TSA for 12 h. No changes in hypodiploid cells were detected after incubation with TSA for 12 h. A slight increase in the hypodiploid cell number was detected in cells treated with 300 nM to 2 µM TSA for 24 h. Hypodiploid cell numbers were dose-dependently increased by treatment with TSA for 48 h. To examine whether TSA-induced tumor cell death requires cytoskeletal dynamics, cells were treated with CytoD or LatB. As shown in Figure 2B and C, CytoD and LatB increased hypodiploid cell formation. Hypodiploid cell numbers were increased from 3 to 18 h after treatment with 1,000 nM CytoD (Figure S1A). LatB, at a concentration of 500 nM, increased hypodiploid cell formation from 3 to 12 h after treatment (Figure. S1B). Interestingly, when cells were treated with TSA in the presence of CytoD or LatB, TSA-induced hypodiploid cell formation was significantly reduced in cells co-treated with 1,000 nM CytoD or 500 nM LatB (Figure 2D). The percentage of hypodiploid cells was 21, 25, and 23%, respectively, in cells treated with 300 nM, 500 nM, and 1,000 nM TSA, compared to ∼7.0% in control cells. By contrast, the hypodiploid cell percentage was 36, 33, and 34%, respectively, in cells treated with 300 nM, 500 nM, and 1,000 nM TSA in the presence of LatB, compared to ∼28% in the LatB-treated non-TSA-treated control group. This demonstrates that the difference in cell death between the TSA-treated group and the control group for 300 nM, 500 nM, and 1,000 nM TSA was reduced from 14, 18, and 16%, respectively, in the absence of LatB to 8, 5, and 6%, respectively, in the presence of LatB. The hypodiploid cell percentage was 22, 24, and 24%, respectively, in cells treated with 300 nM, 500 nM, and 1,000 nM TSA in the presence of CytoD, compared to ∼17.7% in the CytoD-treated non-TSA-treated control group.

Our results were confirmed by staining for annexin V on the apoptotic cell surface and by PI staining. As shown in Figure 2E, the percentage of annexin V-positive cells was 10.4, 12.8, and 12.6%, respectively, in CytoD-, LatB-, and TSA-treated cells, compared to ∼3.3% in the control group. It was increased to about 16.3 and 18.8% in CytoD/TSA- and LatB/TSA-treated cells, respectively. Moreover, our data demonstrate that the difference in percentage of annexin V-positive cells between the TSA-treated group and control cells was reduced from 9.3% in the absence of CytoD or LatB to 5.9 and 6.0% in the presence of Cyto D and LatB, respectively. In addition, the percentage of PI-positive cells was 9.3, 7.9, and 6.5% in CytoD-, LatB-, and TSA-treated cells, respectively, compared to ∼3.6% in control cells. It was increased to about 11.6 and 8.8% in CytoD/TSA- and LatB/TSA-treated cells, respectively. These data demonstrate that the difference in percentage of PI-positive cells between the TSA-treated group and control cells was reduced from 2.9% in the absence of CytoD or LatB to 2.3 and 1.0% in the presence of CytoD and LatB, respectively. This suggests that the difference in cell death between TSA-treated and control cells was reduced by the presence of CytoD, similar to the results for LatB-treated cells.

The same pattern of cell death was observed in DAPI-stained cells (Figure 2F). The percentage of cells with chromosome condensation was 25 and 27% in cells treated with 300 nM and 1,000 nM TSA, respectively, compared to ∼3.0% in control cells. By contrast, the percentage of cells with chromosome condensation was 51 and 59%, respectively, in cells treated with 300 nM and 1,000 nM TSA in the presence of CytoD, compared to ∼45% in the LatB-treated non-TSA-treated control group. This demonstrates that the difference in cell death between TSA-treated and control cells for 300 nM and 1,000 nM TSA was reduced from 22 and 24%, respectively, in the absence of LatB to 6 and 14%, respectively, in the presence of CytoD. In addition, the percentage of cells with chromosome condensation was 50 and 46%, respectively, in cells treated with 300 nM and 1,000 nM TSA in the presence of LatB, compared to ∼34% in the LatB-treated non-TSA-treated control group. This demonstrates that the difference in cell death between TSA-treated and control cells for 300 nM and 1,000 nM TSA was reduced from 22 and 24%, respectively, in the absence of LatB to 16 and 12%, respectively, in the presence of LatB. Collectively, these data suggest that F-actin bundles for dynamic cytoskeleton rearrangement are related to TSA-induced HeLa cell apoptosis.

### The MMP was Decreased by Incubation with CytoD or LatB

**Figure 3:**
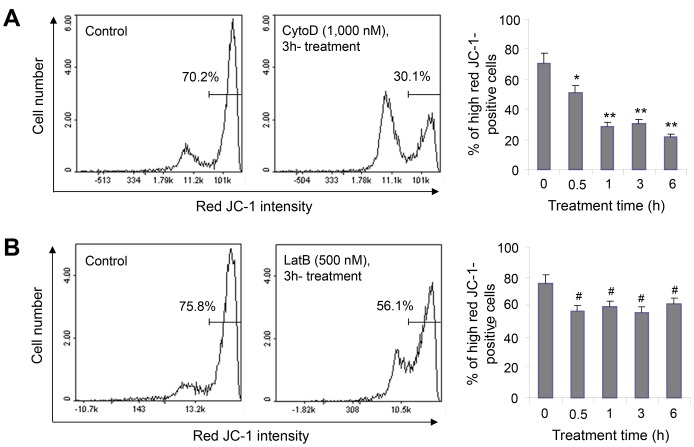
The MMP was decreased by incubation with CytoD or LatB. **A–B:** HeLa cells were treated with 1,000(A) or 500 nM LatB (B) for an appropriate length of time. Cells were detached by incubation with trypsin/EDTA and collected at each time point. A total of 1×10^6^ cells suspended in PBS were incubated with 2.5 µg/ml JC-1 for 10 min at 37°C. Stained cells were washed with PBS twice and analyzed immediately with a NucleoCounter NC-3000 cytometer (ChemoMetec). An MMP collapse was detected as a decrease in the percentage of cells with high red fluorescence intensity. The data in the bar graph represent the mean ± SEM. *p<0.05, **p<0.01 vs. non-CytoD-treated control cells; ^#^ *p*<0.05 vs. non-LatB-treated control cells.

**Figure 4:**
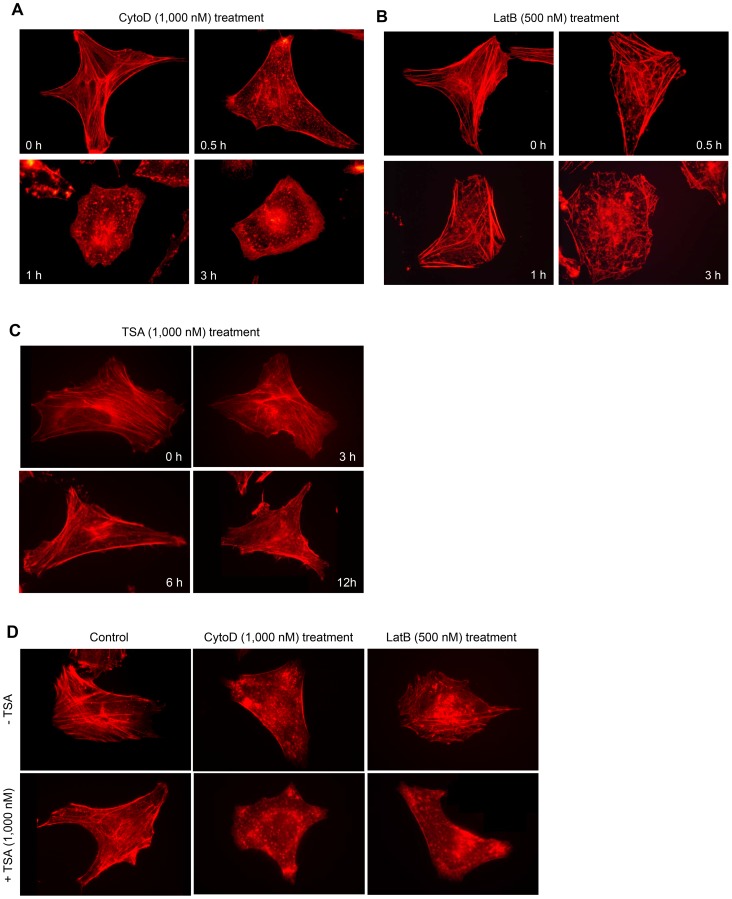
F-actin was disrupted by incubation with CytoD or LatB. **A–C:** HeLa cells were treated with 1,000(A), 500 nM LatB (B), or 1,000 nM TSA (C) for an appropriate amount of time. **D:** HeLa cells were treated for 12 h with 1,000 nM TSA in the absence or presence of CytoD or LatB. Next, the cells were fixed in 3% paraformaldehyde and stained with phalloidin-TRITC diluted 1∶1,000 in sucrose buffer, as described in the Materials and Methods. The cells were then observed under a fluorescence microscope at 1,000× magnification.

Given that mitochondrial dysfunction results in alterations in cellular morphology and adhesion [11], and that mitochondria-actin interactions contribute to cell death [8], we measured the MMP in cells treated with the actin cytoskeleton depolymerizers CytoD and LatB using MitoProbe JC-1 reagent. When HeLa cells were treated with 1,000 nM CytoD for various lengths of time, we found that the MMP gradually decreased between 0.5 and 6 h (Figure 3A). In addition, 500 nM LatB decreased the MMP from 0.5 to 6 h (Figure 3B). The CytoD-treated cells showed no F-actin bundles (Figure 4A). The LatB-treated cells showed a slight decrease in F-actin bundles compared to non-treated control cells (Figure 4B). Only F-actin clumps were visible in 1,000 nM CytoD- and 500 nM LatB-treated cells. Although F-actin bundle formation was disturbed by treatment with CytoD or LatB for 3 h, no changes were detected in basal cell viability (data not shown). In addition, thick F-actin bundles were observed in cells treated with 1,000 nM TSA for 3, 6, or 12 h (Figure 4C). F-actin bundles were decreased while F-actin clumps increased in TSA-treated cells in the presence of CytoD or LatB (Figure 4D). These data might be related to a decrease in TSA-induced apoptosis in the presence of CytoD or LatB (Figure 2), and they suggest that the dynamic rearrangement of F-actin is required for TSA-mediated HeLa cell apoptosis.

### The TSA-induced MMP Collapse was Inhibited by CytoD or LatB Treatment

**Figure 5:**
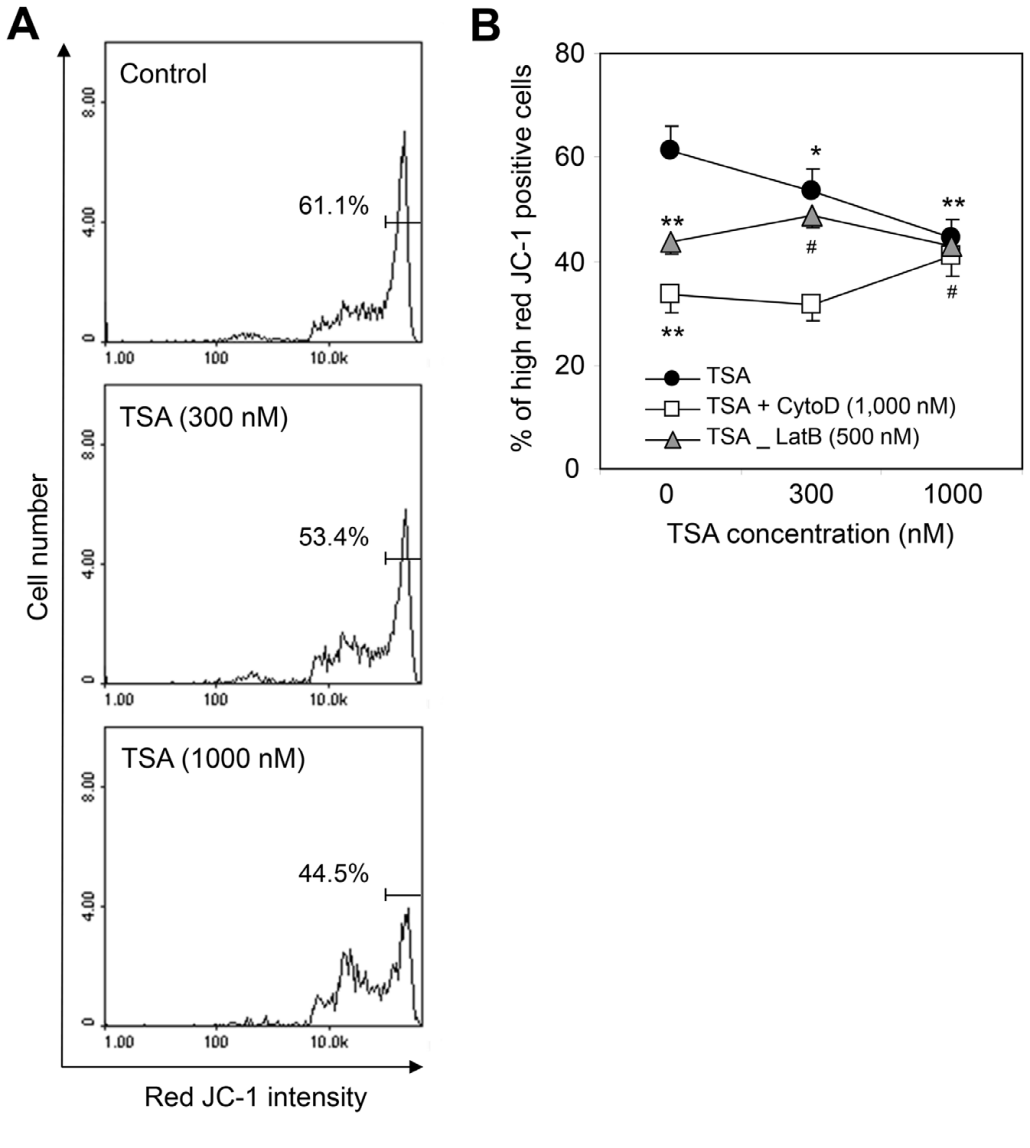
The MMP was decreased by incubation with CytoD or LatB. **A–B:** HeLa cells were treated for 3(•) in the presence or absence of 1,000 nM CytoD (□) or 500 nM LatB (▴). Cells were detached by incubation with trypsin/EDTA and collected at each time point. A total of 1×10^6^ cells suspended in PBS were incubated with 2.5 µg/ml JC-1 for 10 min at 37°C. Stained cells were washed with PBS twice and analyzed immediately with a NucleoCounter NC-3000 cytometer (ChemoMetec). An MMP collapse was detected as a decrease in the percentage of cells with high red fluorescence intensity. The data in the line graph represent the mean ± SEM. *p<0.05 vs. non-TSA-treated and non-CytoD- or non-LatB-treated control cells; ^#^ *p*<0.05 vs. TSA-treated and CytoD- or LatB-treated control cells.

To confirm the effect of cytoskeleton depolymerizers on TSA-induced HeLa cell apoptosis, we examined the MMP in TSA-treated cells in the presence or absence of actin cytoskeleton depolymerizers. The MMP was significantly lowered by treatment with 300 nM or 1,000 nM TSA for 3 h (Figure 5A). However, a small or no difference in MMP between TSA-treated and control cells was detected at each concentration of TSA in the presence of 1,000 nM CytoD or 500 nM LatB, similar to our results for hypodiploid cell formation (Figure 5B). These results demonstrate that the antitumor activity of TSA is low in cells in which the MMP has already been decreased by co-treatment with CytoD or LatB. They also suggest that F-actin bundles and their dynamic rearrangement are necessary for the antitumor activity of TSA involving an MMP collapse. Our findings further suggest that a decrease in actin cytoskeleton dynamics could inhibit the therapeutic effects of antitumor agents by changing the MMP.

## Discussion

Changes in cytoskeleton dynamics may lead to cell death via an apoptosis-like pathway [3]–[5], [12]–[14]. Mitochondrial dysfunction due to mitochondria-actin interactions causes alterations in cellular morphology and adhesion [8], [11]. JasPK, CytoD, and LatB can powerfully alter microfilament dynamics [4], [5]. In mammalian cells, apoptosis is enhanced by treatment with CytoD or JasPK, and by down-regulation of the actin-severing protein gelsolin [6], [7], [15], [16]. By contrast, CytoD attenuates apoptosis in gelsolin-deficient cells [16] and during ischemia-reperfusion [17]. This suggests that cytoskeletal dynamics are involved not only in tumor cell death, but also in drug resistance induction. The resistance of tumor cells to anticancer treatment led to a decrease in survival rate among tumor patients [18]. HDAC inhibitors up-regulate GRP78/BiP, which preserves the MMP after stress and makes cancer cells resistant to HDAC inhibitor-induced apoptosis [20]. We investigated whether drug resistance could be induced by treatment with the antitumor HDAC inhibitor TSA through changes in cytoskeletal dynamics and the MMP. Our data show that TSA and F-actin depolymerizers enhanced apoptotic cell death (Figures 1 and 2) and lowered the MMP (Figures 3 and 5A) in HeLa cells. They also show that the F-actin depolymerizers CytoD and LatB inhibited TSA-induced apoptotic cell death, as judged by an MTT assay and by PI, annexin V, and DAPI staining (Figures 1D and 2D). In addition, these F-actin depolymerizers inhibited an MMP collapse (Figure 5B). This may be explained by a decrease in the sensitivity of tumor cells to TSA due to the prevention of F-actin rearrangement caused by CytoD and LatB treatment. Our data suggest that the development of TSA resistance is induced by changes in cytoskeleton dynamics through consecutive changes in the MMP, even though the detailed mechanism of action of TSA in the actin-mitochondrial interaction remains to be determined.

As the formazan formed in the MTT assay reflects mitochondrial enzyme activity, the absorbance reflects not only cell viability and cell density but also changes in the MMP [22]. In our study, when cells were treated with the same concentration of TSA, the percentage of hypodiploid cells, as assayed by PI staining, was higher than the percent cell viability measured in an MTT assay. Therefore, it is expected that mitochondrial function may play a role in the inhibition of TSA-mediated cell death by co-treatment with cytoskeleton depolymerizers. These data are consistent with those of a previous report showing that actin or actin-binding proteins can influence mitochondrial pathways [8]. Our data indicate that changes in cytoskeleton dynamics may be an additional signaling pathway leading to drug resistance via an MMP collapse.

In previous reports, drug resistance was induced by changes not only in actin microfilaments, but also in microtubules. Class III β-tubulin is a survival factor that directly contributes to drug resistance [23]. While apoptosis in mammalian cells was increased by down-regulation of the actin-severing protein gelsolin [16], CytoD attenuated apoptosis in gelsolin-deficient cells [16]. It is possible that gelsolin levels are reduced by treatment with CytoD or TSA. If that is indeed the case, gelsolin could explain the inhibition of TSA-induced apoptosis in CytoD-co-treated cells. Even though we could not explain all of the phenomena in TSA-induced resistance, our data suggest that complete cytoskeletal structures are required for apoptotic cell death induced by drug treatment. The detailed mechanism of TSA resistance induction remains to be defined. However, our data suggest that maintaining the MMP could be a novel strategy to overcome drug resistance when tumor cells are treated with TSA.

## Supporting Information

Figure S1 **Hypodiploid cell formation by the treatment with CytoD or LatB in the in Hela cells.** **A:** Hela cells were treated with 1,000 nM CytoD for 6, 12, 18 h. **B:** Hela cells were treated with 500 nM LatB for 3, 6, and 12 h. Cells were fixed with 40% ethanol and stained with propidium iodid then analyzed hypodiploid cells with flow cytometry.
(TIF)
