# Supplementary figures and images for: Dynamic Rearrangement of F-Actin Is Required to Maintain the Antitumor Effect of Trichostatin A
Source: PLoS One. 2014 May 20;9(5):e97352. doi: 10.1371/journal.pone.0097352 (PMC4028200; doi:10.1371/journal.pone.0097352)

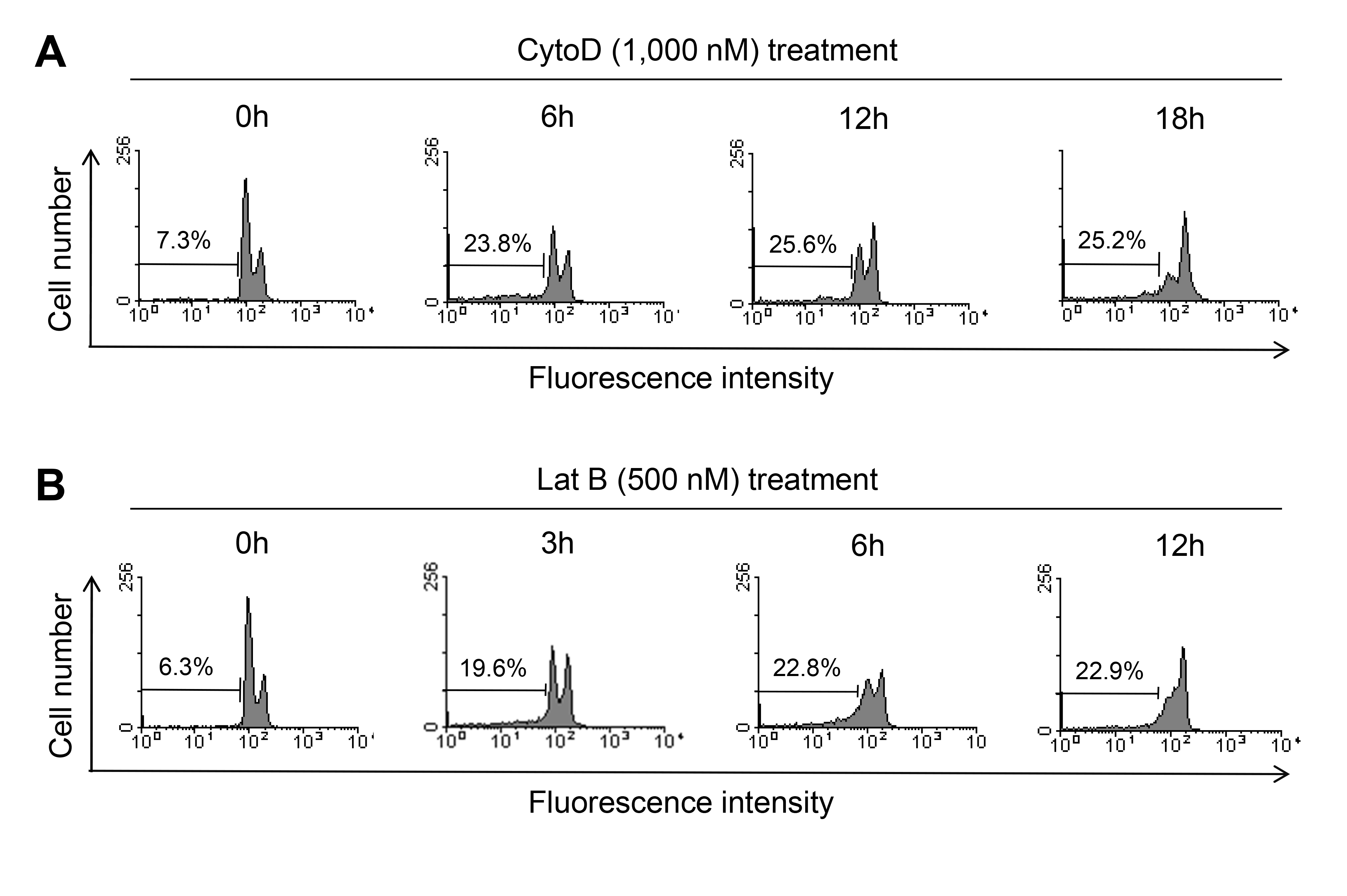

Supplement: Figure S1 — Hypodiploid cell formation by the treatment with CytoD or LatB in the in Hela cells. A: Hela cells were treated with 1,000 nM CytoD for 6, 12, 18 h. B: Hela cells were treated with 500 nM LatB for 3, 6, and 12 h. Cells were fixed with 40% ethanol and stained with propidium iodid then analyzed hypodiploid cells with flow cytometry. (TIF) [file pone.0097352.s001.tif]
